# Impact of COVID-19 convalescence on pregnancy outcomes in patients undergoing IVF/ICSI during fresh ART cycles: a retrospective cohort study

**DOI:** 10.3389/fendo.2023.1298995

**Published:** 2024-01-29

**Authors:** Mingya Cao, Yan Han, Tengfei Feng, Peiyang Lu, Yue Wang, Qingyun Sun, Zhiming Zhao, Wensen Pan

**Affiliations:** ^1^ Department of Reproductive Medicine, The Second Hospital of Hebei Medical University, Shijiazhuang, China; ^2^ Second Department of Respiratory and Critical Care Medicine, The Second Hospital of Hebei Medical University, Shijiazhuang, China

**Keywords:** SARS-CoV-2, COVID-19, fertility, IVF, clinical outcomes

## Abstract

**Objective:**

The aim was to study the impact of coronavirus disease 2019 (COVID-19) convalescence on female fertility and laboratory and clinical outcomes in fresh assisted reproductive technology (ART) cycles.

**Methods:**

In this retrospective cohort study, we analyzed data from 294 patients who had recovered from COVID-19 and who underwent fresh ART cycles between January and March 2023 (COVID-19 group). This group was compared with 631 patients who underwent similar ART cycles in the same period in 2022 but without having been infected with COVID-19 (non-COVID-19 group). The analysis focused on comparison of basic demographic characteristics and laboratory parameters of patients in each group. The primary outcome measure was the clinical pregnancy rate, which was examined to assess the impact of COVID-19 infection on the efficacy of ART treatment.

**Results:**

Basal follicle-stimulating hormone (FSH) levels were significantly lower and antral follicle count (AFC) was markedly higher in the COVID-19 group compared to the non-COVID-19 group (P<0.001 and P=0.004, respectively). The predominant ovarian stimulation protocol in the COVID-19 group was GnRH antagonists (64.85%, P<0.001), with a reduced gonadotropin (Gn) dosage and duration in comparison to the non-COVID-19 group (P<0.05). Although the number of blastocysts formed was lower in the COVID-19 group (P=0.017), this group also exhibited a higher blastocyst freezing rate and a higher rate of high-quality embryos per retrieved oocyte (P<0.001 and P=0.023, respectively). Binary logistic regression analysis indicated that COVID-19 convalescence did not significantly impact clinical pregnancy rates in fresh transfer cycles (odds ratio [OR] = 1.16, 95% confidence interval [CI] = 0.68-1.96, P=0.5874). However, smooth curve-fitting and threshold effect analysis revealed an age-related decline in clinical pregnancy rates in both groups, more pronounced in the COVID-19 group, for women aged over 38 years, with the likelihood of clinical pregnancy decreasing by 53% with each additional year of age (odds ratio [OR] = 0.81, 95% confidence interval [CI] = 0.61–1.08, P=0.1460; odds ratio [OR] = 0.47, 95% CI = 0.21–1.05, P=0.0647).

**Conclusions:**

Our findings present no substantial evidence of adverse effects on clinical pregnancy outcomes in fresh ART cycles in patients undergoing in vitro fertilization (IVF) or intracytoplasmic sperm injection (ICSI) during the period of convalescence from COVID-19. However, age emerges as a significant factor influencing these outcomes. Notably, for women above 38 years of age, the likelihood of clinical pregnancy in patients with a prior COVID-19 infection decreased by 53% with each additional year. This highlights the importance of considering maternal age, especially in the context of COVID-19, when evaluating the likelihood of successful pregnancy following ART treatments.

## Introduction

The coronavirus disease COVID-19 is an infectious disease caused by severe acute respiratory syndrome coronavirus 2 (SARS-CoV-2). Not only can this virus induce severe respiratory disease, but it can also induce multiple histopathological changes in multiple systems and organs, including the kidney (1), brain (2), and liver (3). It utilizes angiotensin-converting enzyme 2 (ACE2) for cell entry (4). ACE2 receptor expression has been identified in the genitourinary organs and the testis (5–8), so the testis (9) and ovary (10) may also be potential target organs for virus infection. In the initial stages of the pandemic, the American Society for Reproductive Medicine (ASRM) issued guidance recommending the suspension of most assisted reproductive technology (ART) treatments, except in the most urgent cases. This recommendation was in line with the guidance provided by the European Society for Human Reproduction and Embryology (11). As a consequence, there has been a significant decline in the number of patients attending infertility clinics over the past three years. Additionally, the majority of patients undergoing *in vitro* fertilization (IVF) or intracytoplasmic sperm injection (ICSI) have opted to either cancel their cycles or freeze oocytes or embryos (12, 13). With the easing of nationwide restrictions relating to coronavirus disease 2019 (COVID-19) since December 2022, reproductive centers are likely to encounter an increasing number of infected patients. The region where our center is situated experienced a concentrated outbreak of COVID-19 between December 2022 and January 2023, providing a reliable opportunity for us to gather pertinent data.

Most previous studies have primarily concentrated on the impact of COVID-19 infection on human reproductive function, particularly focusing on analysis of male semen and the potential detection of COVID-19 mRNA or antibodies in semen (14, 15), follicular fluid, oocytes, endometrial tissue (12, 16, 17), and cervicovaginal secretions (18) of infected patients. In contrast, there is a paucity of literature addressing the specific effects of COVID-19 infection on the pregnancy outcomes of IVF/ICSI procedures. Additionally, two studies have reached opposite conclusions regarding the impact of COVID-19 infection on embryos. Chen et al. report that COVID-19 does not adversely affect oocyte quality or embryo development (19). In contrast, another study posits that previous SARS-CoV-2 infection might influence the developmental potential of embryos (20). Although recent research (21–23) has reported no negative impact of COVID-19 infection on the clinical outcomes of ART treatments, these studies may have limitations due to their small case group sample sizes and the lack of consideration for the impact of the woman’s age on clinical outcomes.

In light of the above, despite the effective control of COVID-19, the pandemic has not been completely eradicated. Sporadic cases continue to occur, and instances of reinfection have been reported. Consequently, studies investigating the impact of COVID-19 on the clinical outcomes of women undergoing ART cycles remain of critical importance.

## Materials and methods

### Study population and design

This retrospective cohort study encompassed all couples infected with COVID-19 who underwent fresh IVF/ICSI treatment cycles at the Reproductive Center of the Second Hospital of Hebei Medical University between January 2023 and March 2023. Women who opted for thawing of frozen oocytes, used donated oocytes or sperm, or were not followed up for clinical outcomes were excluded from the study. Patients were allocated to the COVID-19 group if either member of the couple had been infected with SARS-CoV-2 before oocyte retrieval. It is important to emphasize that all patients included in the study were diagnosed with mild cases of COVID-19. No individuals with moderate or severe symptoms underwent IVF treatment as part of this research. Patients who were not infected with COVID-19 during the same period in 2022 were allocated to the non-COVID-19 group. The diagnosis of COVID-19 infection was confirmed through nucleic acid or antigen testing. Additionally, the interval between recovery time and egg retrieval time was defined as the time from the point at which a patient’s serum SARS-CoV-2 antibody or antigen test was negative to egg retrieval. Patients with COVID-19 infection were followed up until the end of June.

In this study, we recorded demographic characteristics including age, partner’s age, BMI, type and duration of infertility, baseline hormone levels, IVF treatments, and causes of infertility. Additionally, cycle characteristics such as treatment protocol, total gonadotropins (GT) administered, and fertilization method were documented. The primary outcome measure was clinical pregnancy rate, while secondary outcomes included rates of available and high-quality embryos. Given that the varying time intervals between recovery and retrieval and infection status (whether both members or one member of the couple were infected) may influence cycle outcomes differently, we also conducted further subgroup analyses. These analyses assessed the impact of time interval from SARS-CoV-2 recovery to oocyte retrieval and infection status on clinical pregnancy rates. Informed consent was obtained from all subjects prior to their participation in the study. This research was conducted in accordance with the Declaration of Helsinki and received approval from the Ethics Committee of the Second Hospital of Hebei Medical University (2022-R453).

### IVF/ICSI protocols and embryo culture

The controlled ovarian stimulation (COS) protocols carried out at our center were categorized as the gonadotropin-releasing hormone (GnRH) agonist protocol, the GnRH antagonist protocol, the GnRH-a prolonged protocol, or other protocols, including mild stimulation and luteal phase stimulation protocols. The details of COS protocols have been previously presented and thoroughly described (24). For all COS protocols, blood tests and ultrasound were used to monitor hormone levels and follicle growth. When the diameter of the leading follicle reached 18 mm or more than two follicles reached 17 mm, human chorionic gonadotropin (hCG) or GnRH agonists were administered as a trigger. Oocyte retrieval was then performed 36–38 h later.

Oocytes were fertilized through either conventional IVF or ICSI. Pronuclei (PN) were evaluated 16–18 h after insemination. Fertilized oocytes were cultured in G1-plus medium (Gothenburg, Sweden) until day 3, when one or two good-quality embryos were selected for fresh transfer, or cleavage embryos were continued in G2-plus medium until day 5 or day 6; single blastocysts were then transplanted or cryopreserved.

A high-quality embryo (HQE), as evaluated on day 3, was defined as follows: (a) normally fertilized embryo with 4–5 cells on day 2 or 8–10 cells on day 3; (b) <15% fragmentation; (c) uniform blastomeres; (d) absence of multinucleation; (e) absence of zona pellucida defects; (f) absence of perivitelline space granularity; and (g) no inclusions in cytoplasm (25). Blastocyst morphology evaluation was based on the Gardner scoring system (26).

### Outcome assessments

The basic characteristics of the patients were collected, including age, body mass index (BMI), type of infertility, infertility duration, causes of infertility, basal hormone levels, COS protocols used, Gn dosage, Gn duration, and so on; among these, basal FSH, AMH, and AFC were taken to reflect ovarian reserve.

Laboratory outcomes included the number of oocytes retrieved, number and rate of normal fertilizations (2PN), number of cleavages and 2PN cleavages, number and rate of available embryos, number and rate of HQEs on day 3, rate of available embryos per egg, rate of HQEs per egg, number and rate of blastocysts formed, blastocyst freezing rate, number of transferred embryos, and clinical pregnancy rate. The normal fertilization rate was the number of 2PN oocytes divided by the number of oocytes retrieved; the rate of available embryos was the number of available embryos divided by the number of 2PN cleavages; the HQE rate was the number of HQEs at the cleavage stage divided by the number of 2PN cleavages; the blastocyst formation rate was the number of blastocysts divided by the number of day 3 embryos for extended culture; the blastocyst freezing rate was number of frozen blastocysts divided by the number of blastocysts formed; and the whole embryo freezing rate was the number of whole-embryo freezing cycles divided by the number of oocyte retrieval cycles.

For clinical outcomes, the clinical pregnancy rate was the primary outcome measure. The criterion for clinical pregnancy was that 28–30 days after embryo transfer, a gestational sac with heartbeat could be seen in the uterine cavity by transvaginal ultrasound examination.

### Statistical analyses

All statistical analyses were conducted using the SPSS 26.0 software package or EmpowerStats (X&Y solutions, Inc., Boston, MA). Continuous variables are presented as the mean ± SD or median (Q1–Q3). Categorical variables are presented as percentages. For normally distributed variables, analyses of variance and two-independent-sample tests were conducted for group comparisons. For continuous variables following a non-normal distribution, non-parametric Mann–Whitney U tests were employed for group comparisons. Fisher’s exact test or the Chi-square test was performed when comparing categorical variables. Univariate analyses were conducted to identify the possible variables that may affect clinical pregnancy rate. A binary logistic regression analysis was carried out to assess whether COVID-19 infection affects pregnancy outcome in patients undergoing IVF/ICSI. Curve-fitting and threshold effect analyses were conducted to identify non-linear relationships. A p-value <0.05 was considered to indicate statistical significance.

## Results

### Baseline characteristics

**Figure 1:**
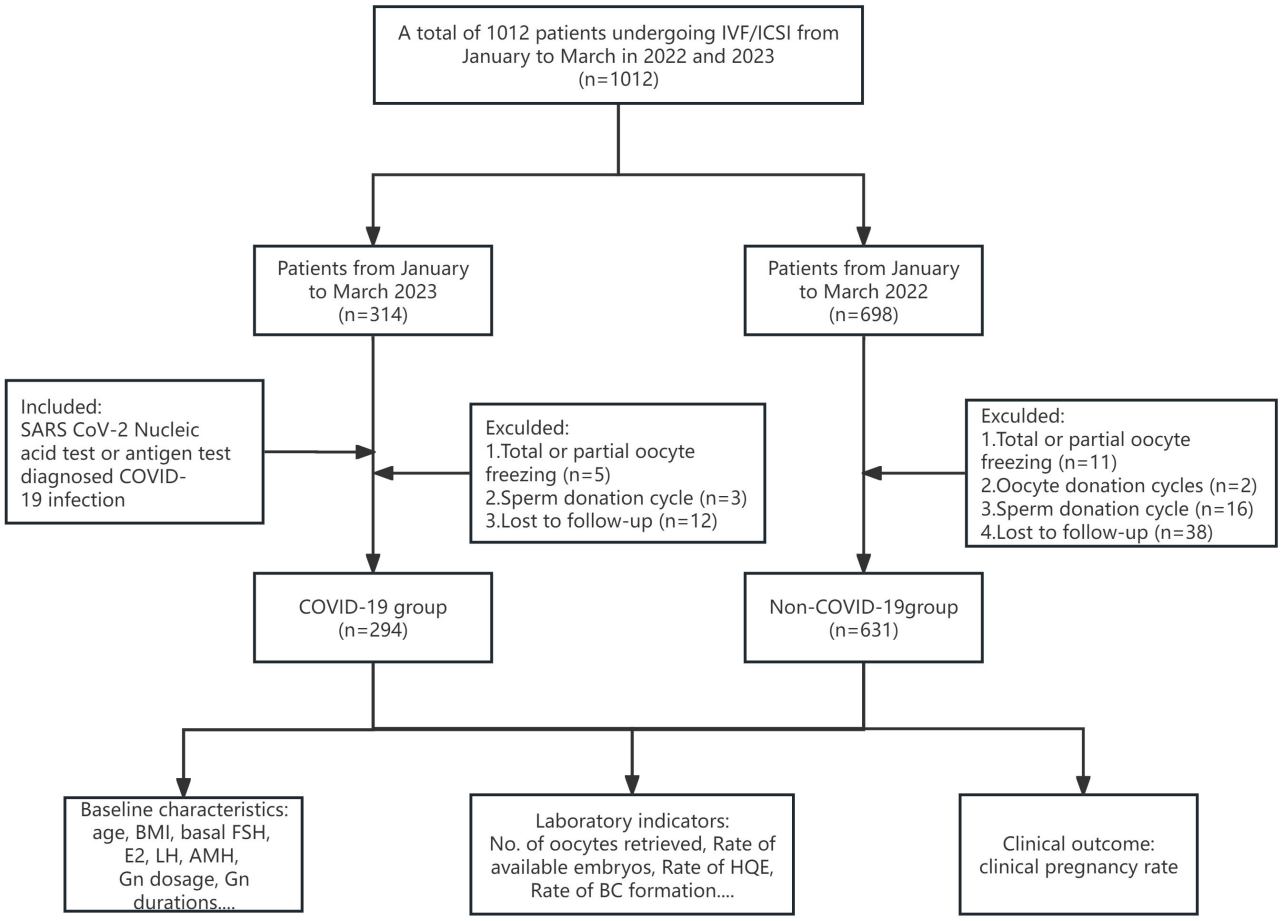
Flowchart of the study. A total of 1,012 couples undergoing IVF/ICSI cycles were enrolled from January to March in 2022 and 2023. After application of the inclusion and exclusion criteria, 925 patients were included in the study. HQE, high quality embryo; BC, blastocyst.

After application of the exclusion criteria, our study included 925 couples. Based on their pre-oocyte-retrieval SARS-CoV-2 infection status, couples were categorized into the COVID-19 group (n=294) or the non-COVID-19 group (n=631), as depicted in **Figure 1**. Among the former group, both partners were infected in the case of 86.05% of the couples, only the female partner was infected in 7.48% of couples, and only the male partner was infected in 6.46% of couples. The baseline characteristics of the patients are presented in **Table 1**. There were no significant differences between the groups in terms of female age, male age, BMI for either sex, basal E2, basal LH, AMH, number of cycles, type or duration of infertility, causes of infertility, fertilization method, semen density, or sperm forward motility rate. However, the basal FSH levels were lower (P<0.001) and the antral follicle count (AFC) was higher (P=0.004) in the COVID-19 group. The predominant controlled ovarian stimulation (COS) protocol in the COVID-19 group was GnRH antagonist (64.85%, P<0.001), and both the gonadotropin (Gn) dosage and duration were significantly lower than those in the non-COVID-19 group (P<0.05).

**Table 1:** Baseline characteristics of patients in the COVID-19 and non-COVID-19 groups.

	COVID-19 (n=294)	Non-COVID-19 (n=631)	Standardized diff.	P-value	P-value*
Age (years)	32.93 ± 5.02	33.09 ± 4.96	0.03 (-0.11, 0.17)	0.636	0.865
Age of male partner (years)	33.64 ± 5.56	33.45 ± 5.35	0.03 (-0.10, 0.17)	0.622	0.477
BMI (kg/m^2^)	24.12 ± 3.97	23.64 ± 3.65	0.13 (-0.01, 0.27)	0.067	0.076
BMI of male partner (kg/m^2^)	25.93 ± 4.22	26.44 ± 4.48	0.12 (-0.02, 0.26)	0.103	0.103
FSH (IU/ml)	7.12 ± 3.57	8.49 ± 4.76	0.33 (0.18, 0.47)	<0.001	<0.001
E2 (pg/ml)	40.59 ± 25.72	45.99 ± 66.42	0.11 (-0.03, 0.25)	0.187	0.58
LH (IU/ml)	5.31 ± 4.25	4.98 ± 5.12	0.07 (-0.07, 0.21)	0.337	0.229
AMH (ng/ml)	3.22 ± 3.07	3.11 ± 3.10	0.04 (-0.10, 0.18)	0.62	0.416
AFC	12.25 ± 8.18	10.68 ± 7.47	0.20 (0.06, 0.34)	0.004	0.006
Cycles			0.10 (-0.04, 0.24)	0.373	–
1	203 (69.05%)	406 (64.34%)			
2	51 (17.35%)	126 (19.97%)			
≥ 3	40 (13.61%)	99 (15.69%)			
Type of infertility			0.07 (-0.07, 0.21)	0.312	–
Primary (%)	142 (48.80%)	284 (45.22%)			
Secondary (%)	149 (51.20%)	344 (54.78%)			
Duration of infertility (years)			0.11 (-0.04, 0.25)	0.349	–
< 1	49 (18.15%)	110 (18.87%)			
1–3	62 (22.96%)	109 (18.70%)			
> 3	159 (58.89%)	364 (62.44%)			
Causes of infertility, n (%)			0.21 (0.08, 0.35)	0.129	–
Tubal factors	117 (39.80%)	236 (37.40%)			
Ovulation disorder	41 (13.95%)	67 (10.62%)			
POR	42 (14.29%)	95 (15.06%)			
EM	14 (4.76%)	60 (9.51%)			
Male factors	31 (10.54%)	76 (12.04%)			
Others	49 (16.67%)	97 (15.37%)			
COS protocols, n (%)			0.34 (0.20, 0.48)	<0.001	–
Antagonist protocol	190 (64.85%)	306 (48.57%)			
Agonist protocol	59 (20.14%)	166 (26.35%)			
GnRH-a prolonged protocol	30 (10.24%)	104 (16.51%)			
Others	14 (4.78%)	54 (8.57%)			
Gn dosage (IU)	2277.45± 903.91	2403.52± 847.32	0.14 (0.00, 0.28)	0.041	0.019
Gn duration (days)	9.22 ± 2.35	9.49 ± 2.59	0.11 (-0.03, 0.25)	0.129	0.002
Fertilization mode, n (%)			0.07 (-0.07, 0.21)	0.336	–
IVF	208 (70.99%)	467 (74.01%)			
ICSI	85 (29.01%)	164 (25.99%)			
Sperm concentration after recovery (10^6^/ml)	56.81 ± 56.15	60.17 ± 58.72	0.06 (-0.08, 0.20)	0.413	0.19
Sperm PR after recovery (%)	33.14 ± 16.39	30.38 ± 16.56	0.17 (0.03, 0.31)	0.022	0.048

### Laboratory indicators and clinical outcomes

**Table 2** presents the laboratory indicators and clinical outcomes of the study participants. Several parameters were comparable between the two groups, including the number of oocytes retrieved, 2PN zygotes, normal fertilization rate, cleavage and 2PN cleavage numbers, available embryos, high-quality embryos (HQEs) on day 3, and blastocyst formation rate. However, a notable difference was observed in the whole-embryo freezing rate, which was significantly higher in the COVID-19 group compared to the non-COVID-19 group (P<0.001). In contrast, the number of blastocyst formations was lower in the COVID-19 group (P=0.017), but the rates of blastocyst freezing and high-quality embryo formation per egg were higher than in the non-COVID-19 group (P<0.001 and P=0.023, respectively). There was no significant difference in clinical pregnancy rate between the groups (51.58% in the COVID-19 group vs. 49.10% in the non-COVID-19 group, P=0.677).

**Table 2:** Laboratory outcomes in the COVID-19 and non-COVID-19 groups.

	COVID-19 (n=294)	non-COVID-19 (n=631)	Standardize diff.	P-value	P-value*
No. of oocytes retrieved	11.19 ± 8.92	10.76 ± 8.57	0.05 (-0.09, 0.19)	0.487	0.681
No. of 2PN zygotes	6.34 ± 5.26	6.51 ± 5.78	0.03 (-0.11, 0.17)	0.671	0.988
Normal fertilization rate (%)	0.62 ± 0.25	0.62 ± 0.25	0.01 (-0.13, 0.15)	0.903	0.772
No. of cleavages	8.15 ± 6.68	8.35 ± 7.02	0.03 (-0.11, 0.17)	0.679	0.732
No. of 2PN cleavages	6.32 ± 5.26	6.46 ± 5.77	0.03 (-0.11, 0.17)	0.709	0.968
No. of available embryos	3.57 ± 3.06	3.35 ± 2.72	0.08 (-0.06, 0.21)	0.272	0.667
Rate of available embryos (%)	0.67 ± 0.42	0.65 ± 0.44	0.05 (-0.09, 0.20)	0.462	0.256
Available embryos per egg (%)	0.40 ± 0.28	0.37 ± 0.26	0.11 (-0.03, 0.25)	0.11	0.185
No. of high-quality embryos (D3)	2.01 ± 2.57	2.13 ± 2.58	0.05 (-0.09, 0.19)	0.504	0.38
High quality embryo rate (D3) (%)	0.31 ± 0.31	0.33 ± 0.29	0.06 (-0.08, 0.20)	0.405	0.156
Quality embryos per egg (%)	0.19 ± 0.21	0.15 ± 0.19	0.21 (0.07, 0.35)	0.003	0.023
No. of blastocysts formed	2.24 ± 3.51	2.62 ± 3.69	0.10 (-0.04, 0.24)	0.148	0.017
Blastocyst formation rate (%)	0.55 ± 0.29	0.57 ± 0.28	0.08 (-0.11, 0.27)	0.419	0.54
Blastocyst freezing rate (%)	0.82 ± 0.31	0.72 ± 0.28	0.32 (0.12, 0.53)	0.002	<0.001
Sperm concentration on OPU day (10^6^/ml)	32.37 ± 12.33	33.17 ± 12.66	0.06 (-0.08, 0.20)	0.37	0.172
Sperm PR on OPU day (%)	23.34 ± 10.64	22.01 ± 10.52	0.13 (-0.01, 0.26)	0.079	0.028
Transferred embryos			0.17 (-0.06, 0.40)	0.169	–
1	14 (14.74%)	59 (21.22%)			
2	81 (85.26%)	219 (78.78%)			
Outcomes			0.28 (0.14, 0.42)	<0.001	–
Whole embryo freezing rate (%)	172 (58.50%)	281 (44.53%)			
Transfer cycle rate (%)	95 (32.31%)	278 (44.06%)			
Cancellation rate (%)	27 (9.18%)	72 (11.41%)			
Clinical pregnancy rate (%)	49 (51.58%)	137 (49.10%)	0.05 (-0.18, 0.28)	0.677	

### The effect of previous COVID-19 infection on clinical pregnancy rate

The results of the univariate analyses are detailed in **Supplementary Table 1**. We conducted a binary logistic regression analysis, adjusting for factors such as couple ages, AMH level, number of cycles, causes of infertility, COS protocol used, fertilization methods, and type and duration of infertility. The analysis revealed that prior COVID-19 infection did not significantly influence the rate of clinical pregnancy in patients undergoing IVF/ICSI treatment (odds ratio [OR] = 1.16, 95% confidence interval [CI] = 0.68–1.96, P=0.5874) (**Table 3**). Similarly, the rates of available embryos and high-quality embryos were also not impacted by COVID-19 infection (results are presented in **Supplementary Table 2**). Furthermore, a sub-analysis of the COVID-19 group under the logistic regression model was conducted based on the time elapsed between COVID-19 recovery and oocyte retrieval, as well as whether one or both members of the couple were infected. In this sub-analysis, after adjusting for factors such as the ages of both partners, BMI, number of cycles, infertility factors, type of infertility, duration of infertility, and fertilization method, we did not find any impact on pregnancy outcomes of the time interval from recovery to oocyte retrieval or whether one or both members of the couple were infected with COVID-19. The detailed results of this sub-analysis are presented in **Supplementary Table 3**.

**Table 3:** The effect of COVID-19 infection on clinical pregnancy rates.

Exposure	Non-adjusted	Adjusted I	Adjusted II
Group (recoded)			
Non-COVID-19	1	1	1
COVID-19	1.10 (0.69, 1.76) 0.6769	1.17 (0.71, 1.94) 0.5333	1.16 (0.68, 1.96) 0.5874

Data presented in the table: β (95% CI) P value/OR (95% CI) P value.

Outcome variable: clinical pregnancy.

Exposure variable: group (recoded).

Non-adjusted: model with no variables adjusted for

Adjusted I: model adjusted for age, age of male partner, FSH, AMH, cycles, and causes of infertility.

Adjusted II: model adjusted for age, age of male partner, AMH, cycles, causes of infertility, COS protocols, fertilization mode, type of infertility, and duration of infertility.

**Figure 2:**
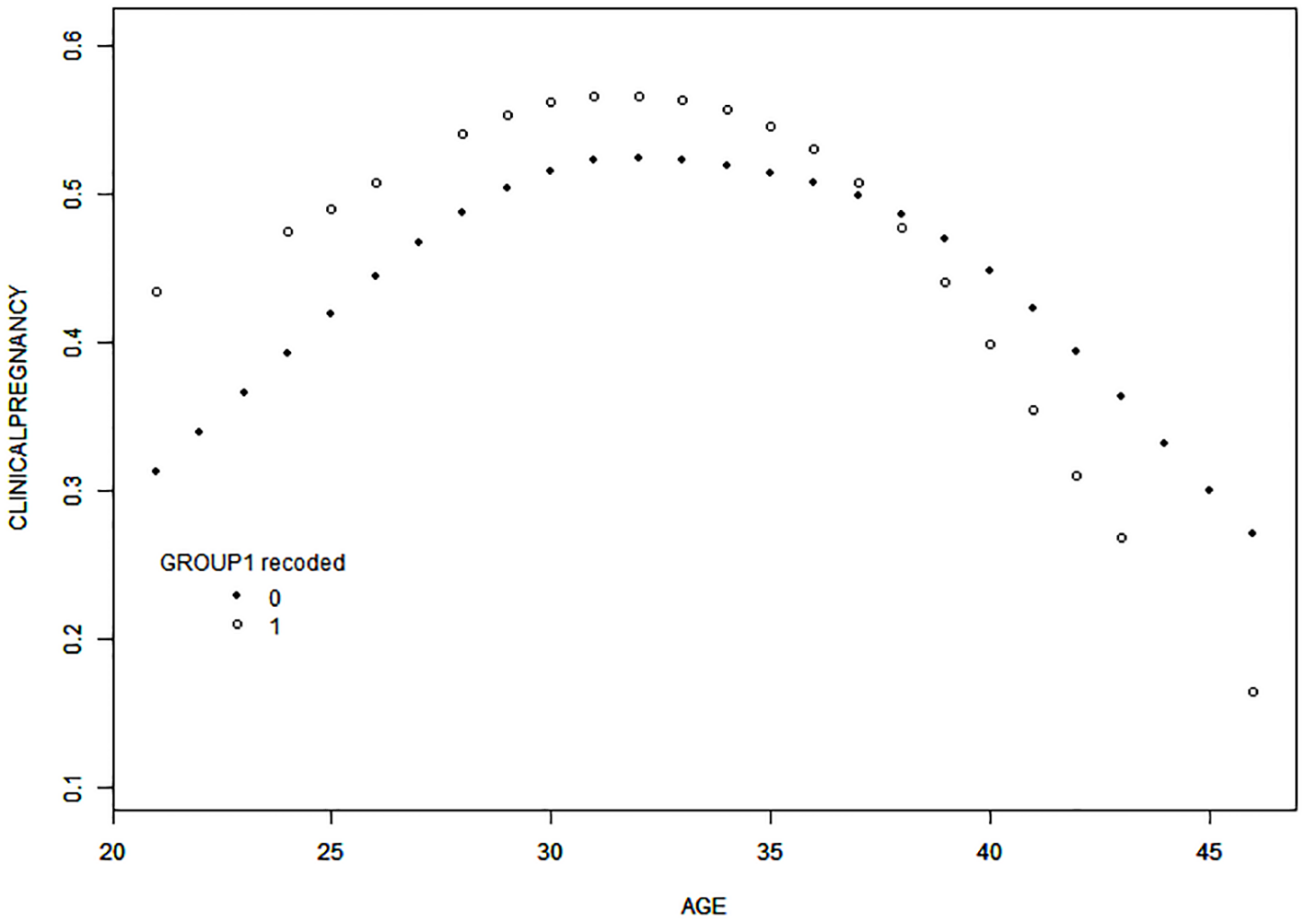
Curve-fitting for the relationship between female age and clinical pregnancy rate. After adjustment for male age, AMH, the number of cycles, causes of infertility, and COS protocols employed, the results of curve-fitting revealed a curvilinear relationship between female age and clinical pregnancy rate in a fresh transplant cycle in both groups. Specifically, the clinical pregnancy rate decreased with increasing age in both groups when female age was > 38 years, and the decrease was more significant in the COVID-19 group. Group 0, non-COVID-19 group; Group 1, COVID-19 group.

Curve-fitting analysis indicated a curvilinear relationship between female age and clinical pregnancy rate in both groups, even after adjusting for male age, AMH level, number of cycles, causes of infertility, and COS protocol used (**Figure 2**). Smooth curve-fitting and threshold effect analysis revealed an age-related decline in clinical pregnancy rate in both groups, which was more pronounced in the COVID-19 group for women aged over 38 years, with the likelihood of clinical pregnancy decreasing by 53% with each additional year of age (odds ratio [OR] = 0.81, 95% confidence interval [CI]= 0.61–1.08, P=0.1460; odds ratio [OR] = 0.47; 95% confidence interval [CI]= 0.21–1.05, P=0.0647) (**Table 4**).

**Table 4:** Threshold effect analysis for age in both groups in terms of impact on clinical pregnancy rate.

Group (recoded)	Non-COVID-19	COVID-19	Overall
Model I			P-interaction: 0.534
One-line effect	1.01 (0.91, 1.12) 0.8631	0.92(0.75, 1.13) 0.4492	1.00 (0.92, 1.09) 0.9576
model II			P-interaction: 0.380
Turning point (K)	38	38	38
< K effect 1	1.04 (0.93, 1.16) 0.4778	1.01 (0.81, 1.26) 0.9168	1.04 (0.95, 1.14) 0.3731
> K effect 2	0.81 (0.61, 1.08) 0.1460	0.47 (0.21, 1.05) 0.0647	0.72 (0.56, 0.94) 0.0160
effect2-1	0.78 (0.58, 1.05) 0.0995	0.46 (0.20, 1.07) 0.0711	0.69 (0.53, 0.91) 0.0094
Model fit value at K	0.10 (-0.37, 0.57)	0.40 (-0.39, 1.20)	0.18 (-0.22, 0.58)
LRT test	0.085	0.029	0.005

Data in the table: β (95% CI) P value/OR (95% CI) P value.

Outcome variable: clinical pregnancy.

Exposure variable: age.

Variables adjusted for: age of male partner, AMH, cycles, causes of infertility, COS protocols.

## Discussion

For the general female population, a history of COVID-19 infection may not adversely affect pregnancy outcomes. However, when focusing on different age groups, the study found that for women over the age of 38, the likelihood of clinical pregnancy significantly decreases with each additional year of age.

Serum levels of FSH, AMH, and basal AFC on days 2–3 of the menstrual period are the three most frequently used and effective markers for ovarian reserve. Kolanska et al. found that mild COVID-19 infection does not alter the ovarian reserve in women treated with ART (27), and similar conclusions were reached in a study by Kahyaoglu et al. (28). In our study, we observed that basal follicle-stimulating hormone (FSH) levels were lower and AFC was higher in the COVID-19 group compared to the non-COVID-19 group, while AMH levels were similar between both groups. This suggests that the data from the population examined during this period do not support the conclusion that COVID-19 infection impacts ovarian reserve function. Furthermore, a higher proportion of patients in the COVID-19 group underwent antagonist protocols; this group was also associated with lower Gn dosage and shorter Gn durations than the non-COVID-19 group. This was likely due to the preference for fast entry cycles and short treatment courses during the pandemic to minimize hospital visits, reduce the risk of nosocomial infection, and improve treatment efficiency. Additionally, a significant decrease in the number of blastocyst formations was observed in the COVID-19 group compared to the non-COVID-19 group; this is in alignment with the findings of Jin Lei et al. (22), who also reported a decrease in blastocyst formation rates following COVID-19 infection. This may be related to the significant co-expression of ACE 2 and TMPRSS2 in the trophoblast ectoderm of late blastocysts, which are more sensitive to SARS-CoV-2 (29). Due to the indeterminate nature of the impact on pregnancy outcome after COVID-19 infection, patients in the COVID-19 group were more likely to be selected for whole-embryo freezing. After adjustment for confounding factors, including the ages of the couple, type and durations of infertility, causes of infertility, and controlled ovarian stimulation (COS) protocol, logistic regression analysis revealed that prior COVID-19 infection did not significantly affect clinical pregnancy rate. These findings are in line with several previous studies that have drawn similar conclusions (30–32).

Age of the woman has a significant impact on embryo quality and pregnancy outcome among patients undergoing assisted conception. In our study, the results of curve-fitting indicated that clinical pregnancy rates were lower among women over the age of 38 in both groups, with the difference being more significant in the COVID-19 group. Under a threshold effect model, the results showed that for women aged over 38 years, the likelihood of clinical pregnancy declined by 53% for every additional year in the COVID-19 group. In order to explore why the clinical outcomes were poorer among older women infected with COVID-19, we compared the data from couples in which the woman was over the age of 38 between the two groups, and found that the available embryo rate, the rate of available embryos per egg, the high-quality embryo rate, the rate of high-quality embryos per egg, the number of blastocysts formed, the blastocyst formation rate, semen parameters after recovery, and sperm concentration on the day of oocyte retrieval were all lower in the COVID-19 group (the results of analysis of these variables are given in **Supplementary Table 4**). Some other studies also have explored potential reasons for the decline in fertility caused by COVID-19. Several studies have reported that oxidative stress plays an important role in COVID-19 infection at the molecular level (33, 34), and the antioxidant system and the accumulation of reactive oxygen species are among the possible reasons for poor pregnancy outcomes. Increased oxidative stress activates the pathogenic mechanism of female fertility (35), alters oocyte epigenetics (36), and ultimately has a negative impact on oocyte quality (37). These two factors may constitute one possible explanation for the more significant decrease in clinical pregnancy rate with advanced age after COVID-19 infection. The challenge of poor pregnancy outcomes in the population of older couples undergoing ART is well-recognized among clinicians, and our study suggests that COVID-19 infection exacerbates these outcomes in this demographic. Consequently, individualized treatment approaches, tailored to the specific needs of this population, are warranted.

Does COVID-19 actually affect gametes? Various studies have provided differing answers. Youngster (19) suggests that COVID-19 infection might have a long-term negative effect on oocyte yield when retrieval occurs more than 180 days after infection. However, a study by Dolgushina presents an opposing view, finding that the parameters of oogenesis and embryogenesis, as well as pregnancy and childbirth rates, did not differ between groups with time intervals of ≤180 days or >180 days (38). In our study, we analyzed the impact of the time interval from recovery to oocyte retrieval on clinical pregnancy rate and found no significant effect. This might be attributed to our study’s time interval range of 24–167 days, which did not extend to 180 days. However, our result was consistent with that of a study by Huang, Jialyu et al. (32), which indicated that prior SARS-CoV-2 infection in females did not adversely affect subsequent IVF treatment, regardless of the time interval following infection. Regarding sperm, another study (39) involving 120 COVID-19 infected subjects found that sperm parameters gradually improved, during convalescence after documented COVID-19 infection from testing an average 53 days after a positive SARS-CoV-2 nasopharyngeal PCR test, suggesting recovery over time following the viral infection.

This study did not include patients undergoing FET, but there are related studies offering insights. Aizer, Adva et al. found that COVID-19 infection did not affect implantation rates or clinical or ongoing pregnancy rates in subsequent FET cycles (31). However, research by Youngster et al. (40) indicated that the clinical pregnancy rate after FET was significantly lower in women infected less than 60 days prior compared to non-infected patients, although there was no significant difference for patients infected more than 60 days prior. This raises the question of whether appropriately delaying pregnancy to allow for normalization of semen parameters, or opting for egg freezing after retrieval or transfer after resuscitation in whole-embryo freezing cycles, could be effective strategies. Additionally, the impact of increased age on clinical outcomes due to delayed assisted reproduction must be considered. Therefore, the optimal time interval before pregnancy and the effectiveness of these methods in improving pregnancy outcomes require further research.

Although larger in terms of sample size than previous research on COVID-19 and pregnancy outcomes, this study has several limitations. Firstly, the potential for infinite statistical differences suggested by the threshold effect analysis indicates the need for an even larger sample size to achieve significant results. Secondly, as this was a single-center retrospective study, the generalizability of our findings is limited, necessitating validation from multi-center global studies. Additionally, only patients in recovery from mild cases of COVID-19 were included, with moderate and severe cases unaccounted for. The short follow-up period also means that any long-term effects on abortion, live birth, and perinatal outcomes remain unknown. Lastly, the lack of data on vaccination status due to the historical nature of the control group is a notable limitation. However, according to the epidemic prevention policies at the time, the majority of the population had been vaccinated, and the current literature (41) suggests that vaccination status does not significantly impact clinical outcomes. In future, we will further trace long-term pregnancy outcomes as well as the health of the offspring, and further conduct multiple subgroup analyses of COVID-19-infected patients, considering variables such as the degree of fever, as well as comparing clinical outcomes between reinfected patients and those infected for the first time, so as to draw more comprehensive and reliable conclusions.

In conclusion, our findings suggest that a history of COVID-19 infection does not have a negative effect on clinical pregnancy rates; however, for women aged over 38 years, the clinical pregnancy rate in fresh transplant cycles was lower in both groups, but especially in the COVID-19 group. Specifically, the likelihood of clinical pregnancy declined by 53% for every additional year of age, indicating that COVID-19 further increases the burden of older age in women undergoing assisted reproductive therapy. In order to solve this problem and to offer reasoned and scientific suggestions or measures, we still need to complete more in-depth research.

## Data availability statement

The datasets presented in this study can be found in online repositories. The names of the repository/repositories and accession number(s) can be found in the article/**Supplementary Material**.

## Ethics statement

The studies involving humans were approved by Ethics Committee of the Second Hospital of Hebei Medical University (2022-R453). The studies were conducted in accordance with the local legislation and institutional requirements. Written informed consent for participation was not required from the participants or the participants’ legal guardians/next of kin in accordance with the national legislation and institutional requirements.

## Author contributions

MC: Data curation, Investigation, Methodology, Writing – original draft. YH: Data curation, Formal analysis, Investigation, Writing – review & editing. TF: Data curation, Software, Writing – review & editing. PL: Investigation, Writing – review & editing, Software. QS: Investigation, Writing – review & editing. YW: Supervision, Writing – review & editing. ZZ: Conceptualization, Writing – review & editing. WP: Writing – review & editing.

## References

[1] Fanelli V Fiorentino M Cantaluppi V Gesualdo L Stallone G Ronco C. Acute kidney injury in SARS-CoV-2 infected patients. Crit Care (2020) 24:155. doi: 10.1186/s13054-020-02872-z 32299479 PMC7161433

[2] Song E Zhang C Israelow B Lu-Culligan A Prado A Skriabine S. Neuroinvasion of SARS-CoV-2 in human and mouse brain. J Exp Med (2021) 218:e20202135. doi: 10.1084/jem.20202135 33433624 PMC7808299

[3] Lei H Ding Y Nie K Dong Y Xu J Yang M. Potential effects of SARS-CoV-2 on the gastrointestinal tract and liver. BioMed Pharmacother (2021) 133:111064. doi: 10.1016/j.biopha.2020.111064 33378966 PMC7700011

[4] Li D Jin M Bao P Zhao W Zhang S. Clinical characteristics and results of semen tests among men with coronavirus disease 2019. JAMA Netw Open (2020) 3:e208292. doi: 10.1001/jamanetworkopen.2020.8292 32379329 PMC7206502

[5] Puliatti S Eissa A Eissa R Amato M Mazzone E Dell’Oglio P. COVID-19 and urology: a comprehensive review of the literature. BJU Int (2020) 125:E7–14. doi: 10.1111/bju.15071 32249538

[6] Li MY Li L Zhang Y Wang XS. Expression of the SARSCoV-2 cell receptor gene ACE2 in a wide variety of human tissues. Infect Dis Poverty (2020) 9:45. doi: 10.1186/s40249-020-00662-x 32345362 PMC7186534

[7] Douglas GC O’Bryan MK Hedger MP Lee DK Yarski MA Smith AI. The novel angiotensin-converting enzyme (ACE) homolog, ACE2, is selectively expressed by adult Leydig cells of the testis. Endocrinology (2004) 145:4703–11. doi: 10.1210/en.2004-0443 15231706

[8] Vishvkarma R Rajender S. Could SARS-CoV-2 affect male fertility? Andrologia (2020) 52:e13712. doi: 10.1111/and.13712 32578263 PMC7361071

[9] Cardona Maya W Du Plessis S Velilla P. SARS-CoV-2 and the testis: similarity with other viruses and routes of infection. Reprod BioMed Online (2020) 40:763–4. doi: 10.1016/j.rbmo.2020.04.009 PMC7162782 32362571

[10] Singh B Gornet M Sims H Kisanga E Knight Z Segars J. Severe acute respiratory syndrome coronavirus 2 (SARS-CoV-2) and its effect on gametogenesis and early pregnancy. Am J Reprod Immunol (2020) 84:e13351. doi: 10.1111/aji.13351 32969123 PMC7537037

[11] Veiga A Gianaroli L Ory S Horton M Feinberg E Penzias A. Assisted reproduction and COVID-19: a joint statement of ASRM, ESHRE and IFFS. Fertil Steril (2020) 114:484–5. doi: 10.1016/j.fertnstert.2020.06.044 PMC7355315 32674808

[12] Boudry L Essahib W Mateizel I Velde Van H Geyter De D Piérard D. Undetectable viral RNA in follicular fluid, cumulus cells, and endometrial tissue samples in SARS-CoV-2-positive women. Fertil Steril (2022) 117(4):771–80. doi: 10.1016/j.fertnstert.2021.12.032 PMC8719925 35272846

[13] Esposito V Rania E Lico D Pedri S Fiorenza A Strati MF. Influence of COVID-19 pandemic on the psychological status of infertile couples. Eur J Obstet Gynecol Reprod Biol (2020) 253:148–53. doi: 10.1016/j.ejogrb.2020.08.025 PMC7443353 32866858

[14] Paoli D Pallotti F Colangelo S Basilico F Mazzuti L Turriziani O. Study of SARS-CoV-2 in semen and urine samples of a volunteer with positive naso-pharyngeal swab. J Endocrinol Invest (2020) 43(12):1819–22. doi: 10.1007/s40618-020-01261-1 PMC7179792 32329026

[15] Corona G Vena W Pizzocaro A Pallotti F Paoli D Rastrelli G. Andrological effects of SARS-Cov-2 infection: a systematic review and meta-analysis. J Endocrinological Invest (2022) 45(12):2207–19. doi: 10.1007/s40618-022-01801-x PMC9080963 35527294

[16] Demirel C Tulek F Celik HG Donmez E Tuysuz G Gökcan B. Failure to detect viral RNA in follicular fluid aspirates from a SARS-CoV-2-positive woman. Reprod Sci (Thousand Oaks Calif.) (2021) 28(8):2144–6. doi: 10.1007/s43032-021-00502-9 PMC7899067 33616884

[17] Barragan M Guillén JJ Martin-Palomino N Rodriguez A Vassena R. Undetectable viral RNA in oocytes from SARS-CoV-2 positive women. Hum Reprod (Oxford England) (2021) 36(2):390–4. doi: 10.1093/humrep/deaa284 PMC7543480 32998162

[18] Agarwal M Basumatary S Bhusan D Pati BK. Detection of severe acute respiratory syndrome corona virus 2 in cervico-vaginal secretion of COVID-19-affected female: A prospective observational study from India. SAGE Open Med (2021) 9:20503121211022993. doi: 10.1177/20503121211022993 34158940 PMC8182207

[19] Chen X Shi H Li C Zhong W Cui L Zhang W. The effect of SARS-CoV-2 infection on human embryo early development: a multicenter prospective cohort study. Sci China. Life Sci (2023) 66(7):1697–700. doi: 10.1007/s11427-023-2291-0 PMC9933004 36795183

[20] Braga DPAF Setti AS Iaconelli A Jr Borges E Jr. Previous infection with SARS-CoV-2 impacts embryo morphokinetics but not clinical outcomes in a time-lapse imaging system. Mol Reprod Dev (2023) 90(1):53–8. doi: 10.1002/mrd.23658 PMC9880701 36576971

[21] Youngster M Avraham S Yaakov O Rabbi ML Gat I Yerushalmi G. IVF under COVID-19: treatment outcomes of fresh ART cycles. Hum Reprod (Oxford England) vol (2022) 37(5):947–53. doi: 10.1093/humrep/deac043 PMC8903458 35212741

[22] Wang M Yang Q Ren X Hu J Li Z Long R. Investigating the impact of asymptomatic or mild SARS-CoV-2 infection on female fertility and in *vitro* fertilization outcomes: A retrospective cohort study. EClinicalMedicine (2021) 38:101013. doi: 10.1016/j.eclinm.2021.101013 34250457 PMC8259363

[23] Levi- Setti PE Cirillo F Immediata V Morenghi E Canevisio V Ronchetti C. First trimester pregnancy outcomes in a large IVF center from the Lombardy County (Italy) during the peak COVID-19 pandemic. Sci Rep (2021) 11(1):16529. doi: 10.1038/s41598-021-96134-9 34400730 PMC8368203

[24] Jiang R Cao M Hao H Jia R Chen P Liu Y. Effects of follicular output rate on cumulative clinical pregnancy rate and cumulative live birth rate in PCOS patients with different characteristics. Front Endocrinol (2022) 13:1079502. doi: 10.3389/fendo.2022.1079502 PMC9806261 36601009

[25] Braga D Setti A Figueira R Iaconelli A Borges E. The importance of the cleavage stage morphology evaluation for blastocyst transfer in patients with good prognosis. J Assist Reprod Genet (2014) 31:1105–10. doi: 10.1007/s10815-014-0266-4 PMC4130936 24893729

[26] Gardner D Schoolcraft W. Culture and transfer of human blastocysts. Curr Opin Obstet Gynecol (1999) 11:307–11. doi: 10.1097/00001703-199906000-00013 10369209

[27] Kolanska K Hours A Jonquière L d'Argent EM Dabi Y Dupont C. Mild COVID-19 infection does not alter the ovarian reserve in women treated with ART. Reprod biomedicine Online vol (2021) 43(6):1117–21. doi: 10.1016/j.rbmo.2021.09.001 PMC8432972 34711516

[28] Kahyaoglu S Ozaksit MG Kahyaoglu I Filiz AA Pekcan MK Atalay E. Does Coronavirus disease-19 infection affect ovarian reserve in infertile women? A retrospective study. J Hum Reprod Sci (2022) 15(4):357–61. doi: 10.4103/jhrs.jhrs_121_22 PMC10077751 37033136

[29] Cheng G-P Guo SM Zhou LQ. Suggestions on cleavage embryo and blastocyst vitrification/transfer based on expression profile of ACE2 and TMPRSS2 in current COVID-19 pandemic. Mol Reprod Dev (2021) 88(3):211–6. doi: 10.1002/mrd.23456 PMC8014618 33624358

[30] Wang M Hu J Huang B Yang Q Liu S Li Z. Investigating the impact of SARS-CoV-2 infection on basic semen parameters and in vitro fertilization/intracytoplasmic sperm injection outcomes: a retrospective cohort study. Reprod Biol Endocrinol RB&E (2022) 20(1):46. doi: 10.1186/s12958-022-00918-1 35260151 PMC8901866

[31] Aizer A Noach-Hirsh M Dratviman-Storobinsky O Nahum R Machtinger R Yung Y. The effect of coronavirus disease 2019 immunity on frozen-thawed embryo transfer cycles outcome. Fertil Steril (2022) 117(5):974–9. doi: 10.1016/j.fertnstert.2022.01.009 PMC8743570 35216833

[32] Huang J Liu Y Xia L Zhao Y Tian L Xu D. Effect of prior female SARS-CoV-2 infection on IVF outcomes: a prospective cohort study. Front Endocrinol (2023) 14:1239903. doi: 10.3389/fendo.2023.1239903 PMC10582695 37859985

[33] Suhail S Zajac J Fossum C Lowater H McCracken C Severson N. Role of oxidative stress on SARS-CoV (SARS) and SARS-CoV-2 (COVID-19) infection: a review. Protein J (2020) 39:644–56. doi: 10.1007/s10930-020-09935-8 PMC7587547 33106987

[34] Delgado-Roche L Mesta F. Oxidative stress as key player in severe acute respiratory syndrome coronavirus (SARS-CoV) infection. Arch Med Res (2020) 51:384–7. doi: 10.1016/j.arcmed.2020.04.019 PMC7190501 32402576

[35] Agarwal A Gupta S Sharma R. Role of oxidative stress in female reproduction. Reprod Biol Endocrinol RB&E (2005) 3:28. doi: 10.1186/1477-7827-3-28 16018814 PMC1215514

[36] Menezo Y Silvestris E Dale B Elder K. Oxidative stress and alterations in DNA methylation: two sides of the same coin in reproduction. Reprod BioMed Online (2016) 33:668–83. doi: 10.1016/j.rbmo.2016.09.006 27742259

[37] Prasad S Tiwari M Pandey A Shrivastav T Chaube S. Impact of stress on oocyte quality and reproductive outcome. J BioMed Sci (2016) 23:36. doi: 10.1186/s12929-016-0253-4 27026099 PMC4812655

[38] Dolgushina NV Menzhinskaya IV Ermakova DM Frankevich NA Vtorushina VV Sukhikh GT. The effect of COVID-19 severity, associated serum autoantibodies and time interval after the disease on the outcomes of fresh oocyte ART cycles in non-vaccinated patients. J Clin Med (2023) 12(13):4370. doi: 10.3390/jcm12134370 37445405 PMC10342348

[39] Donders GGG Bosmans E Reumers J Donders F Jonckheere J Salembier G. Sperm quality and absence of SARS-CoV-2 RNA in semen after COVID-19 infection: a prospective, observational study and validation of the SpermCOVID test. Fertil Steril (2021) 117:287. doi: 10.1016/j.fertnstert.2021.10.022 34937665 PMC8685303

[40] Youngster M Avraham S Yaakov O Rabbi ML Gat I Yerushalmi G. The impact of past COVID-19 infection on pregnancy rates in frozen embryo transfer cycles. J Assisted Reprod Genet (2022) 39(7):1565–70. doi: 10.1007/s10815-022-02517-w PMC9078206 35525900

[41] Albeitawi S Al-Alami ZM Hamadneh J Alqam H Qublan H Natsheh MA. COVID-19 infection and vaccine have no impact on in-vitro fertilization (IVF) outcome. Sci Rep (2022) 12(1):21702. doi: 10.1038/s41598-022-25757-3 36522363 PMC9753879

