# Supplementary material for: Impact of COVID-19 convalescence on pregnancy outcomes in patients undergoing IVF/ICSI during fresh ART cycles: a retrospective cohort study
Source: Front Endocrinol (Lausanne). 2024 Jan 29;14:1298995. doi: 10.3389/fendo.2023.1298995 (PMC10860335; doi:10.3389/fendo.2023.1298995)
Supplement: Supplementary file 1 [file Table_1.doc]

**Supplementary Tables**

**Table 1 The consequences of the univariate analyses**

|  | Statistics | Clinical pregnancy |
| --- | --- | --- |
| Age(years) | 33.04 ± 4.98 | 0.95 (0.91, 1.00) 0.0348 |
| Age(years) | 33.51 ± 5.41 | 0.95 (0.91, 0.99) 0.0205 |
| BMI(kg/m2) | 23.79 ± 3.76 | 1.04 (0.98, 1.10) 0.1992 |
| BMI man (kg/m2) | 26.28 ± 4.40 | 1.01 (0.97, 1.06) 0.5550 |
| Basal FSH (IU/ml) | 8.06 ± 4.46 | 0.97 (0.90, 1.05) 0.4710 |
| Basal LH(IU/ml) | 5.08 ± 4.86 | 1.04 (0.98, 1.12) 0.2072 |
| BasalE2(pg/ml) | 44.26 ± 56.70 | 1.00 (0.99, 1.00) 0.3621 |
| AMH(ng/ml) | 3.14 ± 3.09 | 1.13 (1.02, 1.26) 0.0159 |
| AFC | 11.18 ± 7.73 | 1.02 (0.98, 1.05) 0.3275 |
| Cycles |  |  |
| 1 | 609 (65.84%) | 1.0 |
| 2 | 177 (19.14%) | 1.03 (0.63, 1.70) 0.9016 |
| ≥3 | 139 (15.03%) | 0.32 (0.14, 0.75) 0.0087 |
| Infertility types |  |  |
| Primary(%) | 426 (46.35%) | 1.0 |
| Secondary (%) | 493 (53.65%) | 0.68 (0.45, 1.03) 0.0688 |
| Infertility durations (years) |  |  |
| ＜1 | 159 (18.64%) | 1.0 |
| 1-3 | 171 (20.05%) | 1.13 (0.58, 2.20) 0.7183 |
| ＞3 | 523 (61.31%) | 0.74 (0.42, 1.28) 0.2811 |
| Infertility causes,n(%) |  |  |
| Tubal factors | 353 (38.16%) | 1.0 |
| Ovulation disorder | 108 (11.68%) | 1.00 (0.47, 2.14) 0.9937 |
| POR | 137 (14.81%) | 0.61 (0.32, 1.17) 0.1348 |
| EM | 74 (8.00%) | 0.61 (0.28, 1.31) 0.2012 |
| Male factors | 107 (11.57%) | 1.00 (0.53, 1.90) 0.9988 |
| Others | 146 (15.78%) | 0.81 (0.43, 1.55) 0.5317 |
| COS protocols, n (%) |  |  |
| Antagonist protocol | 496 (53.74%) | 1.0 |
| Agonist protocol | 225 (24.38%) | 1.17 (0.74, 1.87) 0.5025 |
| GnRH-a prolonged protocol | 134 (14.52%) | 0.72 (0.42, 1.26) 0.2571 |
| Others | 68 (7.37%) | 0.00 (0.00, Inf) 0.9798 |
| Gn dosage (IU) | 2363.07 ± 867.38 | 1.00 (1.00, 1.00) 0.3171 |
| Gn durations (days) | 9.41 ± 2.52 | 1.04 (0.95, 1.15) 0.4195 |
| Endometrium thickness (mm) | 11.14 ± 2.36 | 0.96 (0.88, 1.04) 0.3186 |
| Fertilization mode,n(%) |  |  |
| IVF | 675 (73.05%) | 1.0 |
| ICSI | 249 (26.95%) | 0.79 (0.49, 1.29) 0.3500 |
| Transfer embryos |  |  |
| 1 | 73 (19.57%) | 1.0 |
| 2 | 300 (80.43%) | 2.59 (1.50, 4.45) 0.0006 |

Table 2.1 The logistic regression analysis of the consequences of the available embryos rate and the high quality embryos rate

| Exposure | Non-adjusted | Adjust I | Adjust II |
| --- | --- | --- | --- |
| Group recoded |  |  |  |
| non-COVID-19 | 0 | 0 | 0 |
| COVID-19 | 0.02 (-0.04, 0.09) 0.4622 | 0.02 (-0.04, 0.08) 0.4755 | 0.01 (-0.05, 0.07) 0.7199 |

Data in the table: β (95%CI) P value / OR (95%CI) P value

outcome variable: Available embryos rate

Exposure variable: Group recoded

Non-adjusted model adjust for: None

Adjust I model adjust for: Age; Ageman; AMH; Cycles; Infertility causes.

Adjust II model adjust for: Age; Ageman; AMH; Cycles; Infertility causes; COS protocols.

Table 2.2 The logistic regression analysis of the consequences of the available embryos rate and the high quality embryos rate

| Exposure | Non-adjusted | Adjust I | Adjust II |
| --- | --- | --- | --- |
| Group |  |  |  |
| non-COVID-19 | 0 | 0 | 0 |
| COVID-19 | -0.02(-0.06,0.02) 0.4047 | -0.02 (-0.06, 0.03) 0.4288 | -0.02(-0.06, 0.03) 0.4371 |

Data in the table: β (95%CI) P value / OR (95%CI) P value

outcome variable: High quality embryos rate

Exposure variable: Group recoded

Non-adjusted model adjust for: None

Adjust I model adjust for: Age; Ageman; AMH; Cycles; Infertility causes.

Adjust II model adjust for: Age; Ageman; AMH; Cycles; Infertility causes; COS protocols.

Table 3.1 Clinical pregnancy rates with different time intervals between recovery time and oocyte retrieval time. (time intervals as a continuous variable)

| Exposure | Adjust I | Adjust II |
| --- | --- | --- |
| Recovery intervals | 0.99 (0.98, 1.01) 0.3254 | 0.99 (0.98, 1.01) 0.3993 |

Data in the table：β (95%CI) P value / OR (95%CI) P value 
outcome variable: Clinical pregnancy
Exposure variable: recovery intervals
Adjust I model adjust for: Age; Ageman; BMI; Cycles; Infertility causes.
Adjust II model adjust for: Age; Ageman; BMI; AMH; Cycles; Infertility causes; COS protocols; Infertility types; Infertility durations; Fertilization mode. 

Table 3.2 Clinical pregnancy rates with different time intervals between recovery time and oocyte retrieval time. (time intervals as a categorical variable)

| Exposure | Adjust I | Adjust II |
| --- | --- | --- |
| Recovery intervals |  |  |
| ＜60 days | 1.0 | 1.0 |
| 60-90 days | 1.10 (0.49, 2.48) 0.8233 | 1.14 (0.47, 2.76) 0.7777 |
| ＞90days | 1.35 (0.60, 3.05) 0.4659 | 0.90 (0.37, 2.20) 0.8094 |

Data in the table：β (95%CI) P value / OR (95%CI) P value 
outcome variable: Clinical pregnancy
Exposure variable: Recovery intervals
Adjust I model adjust for: Age; Ageman; BMI; Cycles; Infertility causes.
Adjust II model adjust for: Age; Ageman; BMI; AMH; Cycles; Infertility causes; COS protocols; Infertility types; Infertility durations; Fertilization mode. 

Table 3.2 Clinical pregnancy rates with the infection status (whether both or one member of a couple were infected)

| Exposure | Adjust I | Adjust II |
| --- | --- | --- |
| Infection status |  |  |
| Only the female partner infected | 1.0 | 1.0 |
| Only the male partner infected | 0.26 (0.04, 1.74) 0.1654 | 0.19 (0.02, 2.18) 0.1819 |
| Both spouses infected | 0.62 (0.18, 2.11) 0.4412 | 0.44 (0.10, 1.96) 0.2830 |

Data in the table：β (95%CI) P value / OR (95%CI) P value 
outcome variable: Clinical pregnancy
Exposure variable: Infection status
Adjust I model adjust for: Age; Ageman; BMI; Cycles; Infertility causes.
Adjust II model adjust for: Age; Ageman; BMI; AMH; Cycles; Infertility causes; COS protocols; Infertility types; Infertility durations; Fertilization mode. 
 

Table 4 Comparison of data on patients with female age> 38 years between two groups

|  | COVID-19  n=44 | non-COVID-19  n=87 | Standardize diff. | P-value | P-value* |
| --- | --- | --- | --- | --- | --- |
| Age(year) | 40.95 ± 2.13 | 42.07 ± 2.29 | 0.50 (0.14, 0.87) | 0.008 | 0.004 |
| Age man(year) | 41.64 ± 4.92 | 42.06 ± 4.91 | 0.09 (-0.28, 0.45) | 0.644 | 0.416 |
| BMI(kg/m^2^) | 23.68 ± 3.61 | 23.58 ± 3.20 | 0.03 (-0.33, 0.39) | 0.866 | 0.918 |
| BMIman(kg/m^2^) | 25.91 ± 3.88 | 25.58 ± 3.75 | 0.09 (-0.28, 0.46) | 0.641 | 0.941 |
| FSH (IU/ml) | 9.39 ± 5.29 | 11.69 ± 7.01 | 0.37 (-0.00, 0.74) | 0.063 | 0.031 |
| E2(pg/ml) | 46.64 ± 32.03 | 55.11 ± 69.50 | 0.16 (-0.21, 0.53) | 0.455 | 0.963 |
| LH(IU/ml) | 5.47 ± 4.45 | 5.23 ± 10.05 | 0.03 (-0.34, 0.40) | 0.883 | 0.116 |
| AMH(ng/ml) | 1.44 ± 1.33 | 1.20 ± 1.50 | 0.17 (-0.20, 0.54) | 0.384 | 0.075 |
| AFC | 7.00 ± 5.60 | 5.63 ± 4.35 | 0.27 (-0.09, 0.64) | 0.126 | 0.206 |
| Gn dosage(IU) | 2644.19 ± 1062.24 | 2582.75 ± 1095.42 | 0.06 (-0.31, 0.43) | 0.765 | 0.681 |
| Gn durations(days) | 8.73 ± 3.16 | 7.95 ± 3.87 | 0.22 (-0.14, 0.58) | 0.254 | 0.214 |
| No. of oocytes retrieved | 5.64 ± 5.28 | 4.07 ± 4.20 | 0.33 (-0.04, 0.69) | 0.067 | 0.04 |
| No. of 2PN zygotes | 3.36 ± 3.64 | 2.64 ± 3.12 | 0.21 (-0.15, 0.58) | 0.241 | 0.181 |
| Normal fertilization rate (%) | 0.67 ± 0.28 | 0.68 ± 0.38 | 0.02 (-0.37, 0.41) | 0.931 | 0.973 |
| No. of cleavage | 4.32 ± 4.11 | 3.32 ± 3.63 | 0.26 (-0.11, 0.62) | 0.159 | 0.117 |
| No. of 2PN cleavage | 3.32 ± 3.65 | 2.63 ± 3.10 | 0.20 (-0.16, 0.57) | 0.263 | 0.231 |
| No. of available embryos | 1.91 ± 1.36 | 1.71 ± 1.68 | 0.13 (-0.23, 0.49) | 0.504 | 0.228 |
| Available embryos rate(%) | 0.71 ± 0.41 | 0.79 ± 0.47 | 0.18 (-0.22, 0.59) | 0.382 | 0.538 |
| Available embryos per egg rate(%) | 0.44 ± 0.33 | 0.52 ± 0.39 | 0.21 (-0.17, 0.58) | 0.291 | 0.367 |
| No. of high quality embryos(D3) | 1.00 ± 1.43 | 1.06 ± 1.62 | 0.04 (-0.33, 0.41) | 0.841 | 0.961 |
| High quality embryos rate(D3)(%) | 0.29 ± 0.34 | 0.37 ± 0.37 | 0.25 (-0.15, 0.65) | 0.233 | 0.241 |
| Quality embryos per egg rate(%) | 0.17 ± 0.23 | 0.20 ± 0.29 | 0.12 (-0.26, 0.50) | 0.548 | 0.733 |
| No. of form blastocyst | 0.61 ± 1.35 | 0.63 ± 1.53 | 0.01 (-0.35, 0.38) | 0.946 | 0.9 |
| Blastocyst formation rate (%) | 0.43 ± 0.34 | 0.46 ± 0.35 | 0.09 (-0.53, 0.71) | 0.771 | 0.733 |
| Blastocyst freezing rate (%) | 0.79 ± 0.34 | 0.77 ± 0.33 | 0.06 (-0.65, 0.78) | 0.861 | 0.882 |
| Sperm concentration after recovery(10^6^/ml) | 52.54 ± 46.96 | 61.52 ± 54.53 | 0.18 (-0.19, 0.54) | 0.358 | 0.35 |
| Sperm PR after recovery(%) | 30.20 ± 15.12 | 30.74 ± 16.76 | 0.03 (-0.34, 0.41) | 0.861 | 0.694 |
| Sperm concentration on OPU day((10^6^/ml)) | 33.43 ± 13.01 | 35.71 ± 10.73 | 0.19 (-0.18, 0.56) | 0.299 | 0.384 |
| Sperm PR on OPU day(%) | 24.52 ± 10.36 | 24.47 ± 10.10 | 0.01 (-0.36, 0.37) | 0.977 | 0.706 |
| Cycles |  |  | 0.11 (-0.26, 0.47) | 0.853 | - |
| 1 | 22 (50.00%) | 42 (48.28%) |  |  |  |
| 2 | 5 (11.36%) | 13 (14.94%) |  |  |  |
| ≥3 | 17 (38.64%) | 32 (36.78%) |  |  |  |
| Infertility types |  |  | 0.17 (-0.19, 0.54) | 0.341 | - |
| Primary(%) | 9 (20.45%) | 12 (13.95%) |  |  |  |
| Secondary (%) | 35 (79.55%) | 74 (86.05%) |  |  |  |
| Infertility durations(years) |  |  | 0.35 (-0.04, 0.74) | 0.188 | - |
| ＜1 | 7 (18.42%) | 19 (24.36%) |  |  |  |
| 1-3 | 10 (26.32%) | 10 (12.82%) |  |  |  |
| ＞3 | 21 (55.26%) | 49 (62.82%) |  |  |  |
| Infertility causes,n(%) |  |  | 0.17 (-0.19, 0.53) | 0.972 | - |
| Tubal factors | 18 (40.91%) | 31 (35.63%) |  |  |  |
| Ovulation disorder | 1 (2.27%) | 1 (1.15%) |  |  |  |
| POR | 15 (34.09%) | 35 (40.23%) |  |  |  |
| EM | 3 (6.82%) | 6 (6.90%) |  |  |  |
| Male factors | 2 (4.55%) | 3 (3.45%) |  |  |  |
| Others | 5 (11.36%) | 11 (12.64%) |  |  |  |
| COS protocols, n (%) |  |  | 0.36 (-0.01, 0.72) | 0.333 | - |
| Antagonist protocol | 35 (79.55%) | 58 (66.67%) |  |  |  |
| Agonist protocol | 2 (4.55%) | 4 (4.60%) |  |  |  |
| GnRH-a prolonged protocol | 1 (2.27%) | 1 (1.15%) |  |  |  |
| Others | 6 (13.64%) | 24 (27.59%) |  |  |  |
| Fertilization mode,n(%) |  |  | 0.27 (-0.09, 0.64) | 0.13 | - |
| IVF | 32 (72.73%) | 73 (83.91%) |  |  |  |
| ICSI | 12 (27.27%) | 14 (16.09%) |  |  |  |
| Transfer embryos |  |  | 0.20 (-0.47, 0.86) | 0.561 | - |
| 1 | 5 (41.67%) | 10 (32.26%) |  |  |  |
| 2 | 7 (58.33%) | 21 (67.74%) |  |  |  |
| Outcome |  |  | 0.37 (0.00, 0.73) | 0.148 | - |
| Whole embryo freezing rate(%) | 25 (56.82%) | 34 (39.08%) |  |  |  |
| Transfer cycle rate(%) | 12 (27.27%) | 31 (35.63%) |  |  |  |
| Cancellation rate(%) | 7 (15.91%) | 22 (25.29%) |  |  |  |
